# Level of interleukin 17 in inflammatory bowel disease and its relation with disease activity

**DOI:** 10.1186/s12876-024-03218-7

**Published:** 2024-04-15

**Authors:** Ayman Menesy, Mohamed Hammad, Salah Aref, Fatma Adel Mourad Abozeid

**Affiliations:** 1 Professor of Internal Medicine, Hepatology and Gastroenterology, Faculty of Medicine, Mansoura University, Mansoura, Egypt; 2 Resident of Hepatology and Gastroenterology, Mansoura University, Mansoura, Egypt; 3 Professor of clinical pathology, faculty of medicine, Mansoura University, Mansoura, Egypt; 4 Lecturer of Internal Medicine, Hepatology and gastroenterology, Faculty of Medicine, Mansoura University, Mansoura, Egypt; 5 Mansoura Specialized Medical Hospital, Mansoura University, Mansoura, Egypt

**Keywords:** Interleukin 17, IBD, Markers of activity

## Abstract

**Background:**

Inflammatory bowel disease (IBD) is a chronic relapsing inflammatory disorder of the gastrointestinal tract (GIT).It results in progressive intestinal epithelium structural and functional damage that necessitates lifetime medication.Thereis imbalance in the production of T helper 1 (Th1), Th2 and Th17 cytokines. This plays a crucial role in the chronic inflammatory process and the defective immune response to pathogenic agents; thus promoting the recurrence of the disease.Our aim of this study was to detect serum IL-17 levels in IBD patients and its relation with disease activity.

**Methods:**

This was a single center case control study, conducted at hepatology and gastroenterology unit, Mansoura specialized Medical Hospital, Egypt.Patients who were included were aged 18–65 years, diagnosed either Ulcerative Colitis (UC)or Crohn’s Disease (CD) based on previous colonoscopy.IBD activity was measured for UC using the MAYO score and CD using the CD activity index (CDAI). Fifty five patients were UC, 24 patients were CD, 21 patients were control.Patients who were excluded were under 15 years old, with history of GIT malignancy, or any serious comorbidities. Study protocol was approved by Institution Research Board (IRB) of Mansoura Medical College.All patients were subjected to full history taking, routine physical examination, colonoscopy and laboratory investigations including serum IL-17 levels by ELISA besides CBC, CRP, ESR and fecal calprotectin.

**Results:**

Serum IL-17 level was increased significantly among UC; median (min-max) = 72(21–502)pg/ml, in CD 54.5(25–260) versus control 19 (14–35), *P* < 0.001.However, it was not correlated to the disease activity either Mayo score of UC or CDAI of CD.There was significant correlation to the extent of inflammation in UC affecting the colon (either proctosigmoiditis, left sided colitis or pan colitis), also to the type of CD (either inflammatory, stricturing or fistulizing) by *P* < 0.05.It was not correlated significantly with any of the IBD activity markers (CRP, ESR, or fecal calprotectin).Yet there was negative significant correlation with Hb level (*r* =-0.28, *p* = 0.005).There was not significant association between median serum level of IL-17 & duration of disease (*P* = 0.6).However, median IL-17 was higher among hospitalized cases than non-hospitalized (73 & 55, pg/ml respectively; *p* < 0.002). AUC was significantly differentiating between IBD and control group = 0.993 with the best-detected cut off point from curve 32 pg/ml yielding sensitivity of 97.5% and specificity of 95.2%.

**Conclusion:**

Serum IL-17 increases in colonic inflammation significantly more than in control group, however its increase is not correlated to IBD activity.

## Introduction

Inflammatory bowel disease (IBD)is a chronic relapsing inflammatory disorder of the gastrointestinal tract (GIT). Multiple factors including immunological abnormality, genetic factors, dietary changes, and increased antimicrobial exposure affecting host–microbial homeostasis have been linked to an increase in the prevalence of IBD. It is classified into two major subtypes based on pathological features and disease manifestation, Ulcerative Colitis (UC) and Crohn’s Disease (CD) [1].

Chronic inflammation in IBD is characterized by an imbalance in the production of Th1, Th2 and Th17 cytokines.This imbalance of cytokine profile is important in chronic inflammatory process. The lack of appropriate regulation of T cells and over-production of effector T cells are involved in the development and exacerbation of IBD. It has been demonstrated that immune cells secrete active products that are associated with the initiation and maintenance of inflammation and result in damage of gut tissue. Further, altered regulations of several cytokines have been implicated in the pathogenesis of UC and CD. Among these cytokines are interleukin (IL) − 17 [2].

IL-17 has been implicated in several inflammatory disorders, such as rheumatoid arthritis, multiple sclerosis, systemic sclerosis, systemic lupus erythematosus, psoriasis, bronchial asthma, renal allograft rejection, and ankylosing spondylitis. However, the pathophysiological role of IL-17 in IBD remains unclear [3].

IL-17 is a pro-inflammatory cytokine which is involved in triggering strong immune responses during chronic inflammation, It is a signature cytokine of T helper 17 (Th17) cells, therefore, a pathogenic role for IL-17 in IBD has been suggested. Many previous reports found that serum IL-17 levels were altered according to disease activity.The usefulness of serum IL-17 in estimating disease activity of IBD patients remains unclear [4].So our aim of study wasto detect serum IL-17 levels in IBD patients and evaluate its relationship with disease activity.

## Methodology

Type of study: this was a cross-sectional case control study that was carried out on 100 subjects who attended IBD Clinic at specialized Medical Hospital, Mansoura University, EGYPT.

Subjects selection: total number of 100 subjects (79 IBD patients and 21 healthy controls) were included in the study.Patients were classified into three groups: first groupincluded 55 patients with UC, second groupincluded 24 patients with CDand the third group included 21 healthy volunteers as a control group.

Inclusion criteria: patients included were both sexes (male or female), diagnosed as IBD patients (UC or CD) based on ECCO guidelines 2019.They wereaged from 18 to 65 years.Patients may be “active” or “inactive”according to patients reported symptoms, elevated inflammatory markers, endoscopic assessment and histology scores. Patients may be newly diagnosed or on existing therapy including 5 amino salicylates (oral or topical), azathioprine, corticosteroids (oral or systemic) and biological treatment (anti TNF or anti-interleukin IL-12/23).

Exclusion criteria: patients with malignant condition as colorectal cancer or surgical resection of colon, or other comorbidities as; liver cell failure, chronic renal failure, heart failure or complicated diabetes mellitus.Inability to or unwillingness to undergo flexible sigmoidoscopy or colonoscopy were excluded.

IBD activity was measured for UC using the MAYO score [5] and CD using the CD activity index (CDAI) [6]. Patients who had MAYO score of ≥ 1, or CDAI ≥ 150 were considered active patients. While those of MAYO ˂ 1 and CDAI ˂150 were considered inactive.

Sample size calculation for this case control study was based on correlation between IL-17 and IBD activity retrieved from previous researches [7, 12].Through comparing the mean of IL-17 between the 3 groups (UC, CD and control) by one way anova and depending on Spearman correlation between IL-17 and IBD activity, sample size calculation was based on correlation co-efficient using the following formula ***Hulley et al., 2013*** [8].Total sample size = N = [(Z_α_+Z_β_)/C]^2^ + 3 = 95.The standard normal deviate for α = Z_α_ = 1.9600.The standard normal deviate for β = Z_β_ = 1.2816.C = 0.5 * ln[(1 + r)/(1-r)] = 0.3372.

All patients and control group were subjected to through history takingand full clinical examination.Some laboratory investigationswere done including: complete blood count (CBC), Erythrocyte Sedimentation Rate (ESR), C-reactive protein (CRP)byCobas C311 of blood sample.Fecal calprotectin was done by testing stool sample usingenzyme-linked immunosorbent assays (ELISA), then measured as mcg/g (˂ 50 is considered to be normal, between 50 and 100 was coupled with digestive symptoms as IBS, between 100 and 250 is inconclusive, and ˃ 250 means IBD is likely).

Serology of serum Interleukin 17:(IL-17) measurements were performed using conventional enzyme-linked immunosorbent assays (ELISA)method for human IL-17. Three milliliters of venous blood were extracted from cases and control group and centrifuged for 15 min at 3000 rpm. Aliquots were then taken and stored at -80 °C until tests were performed.Human Interleukin 17 (IL-17) ELISA kit, Version Cat. No E0142HU was used to detect level of IL17 in subjects sera according to the manufacturer’s instructions. Raw data were acquired using Mars Data Analysis Software for quantification (pg/mL). A 4-Parameter fit based calibration curve was generated using known concentrations of the protein standard following the protocol. IL-17 concentrations were obtained.

Endoscopy: sigmoidoscopy and/orileo-colonoscopy was done only for cases.It was performed after good preparation of colon in the endoscopy unit of Mansoura Specialized Medical Hospital using colonoscopy Pentax PK 100 Video scope. Preparation for colonoscopy was done by 2–4 L of hypertonic polyethylene glycol.The cleaning procedure started 24-hour prior to the procedure. Propofol IVwas used for sedation at the patient’s request. All biopsies were examined by a pathologist, from Department of Pathology after it was embedded in paraffin and fixed in 10% formalin solution, then were stained by hematoxylin and eosin.

Statistical analysis: IBM SPSS software package version 25.0 was used to establish data analysis. Qualitative data were described using number and percent. Quantitative data were described using median (minimum and maximum) (interquartile range) for non - normally distributed data after testing normality using Kolmogrov - Smirnov test. All tests were 2 - tailed. Non parametric tests; Mann Whitney U test was used to compare between 2 groups, while Kruskal Wallis between more than 2 studied independent groups.Spearman correlation coefficient was used to correlate between continuous non normally distributed data. Probability value (P values) of less than 0.05 were considered statistically significant. Diagnostic accuracy of qualitative tests was obtained using Receiver Operating Characteristics (ROC) curve to detect the cutoff with best sensitivity and specificity.

The study was approved by the Ethical Committee in Faculty of Medicine, Mansoura University. Code number: MS.20.10.22.

## Results

### Descriptive data of all subjects

The current study included 100 subjects. 79 patients were with inflammatory bowel disease (55 UC and 24 CD) and 21were healthy control.71% of subjects were under 40 years old, 54% were males and 56% were from urban areas.

**Fig. 1:**
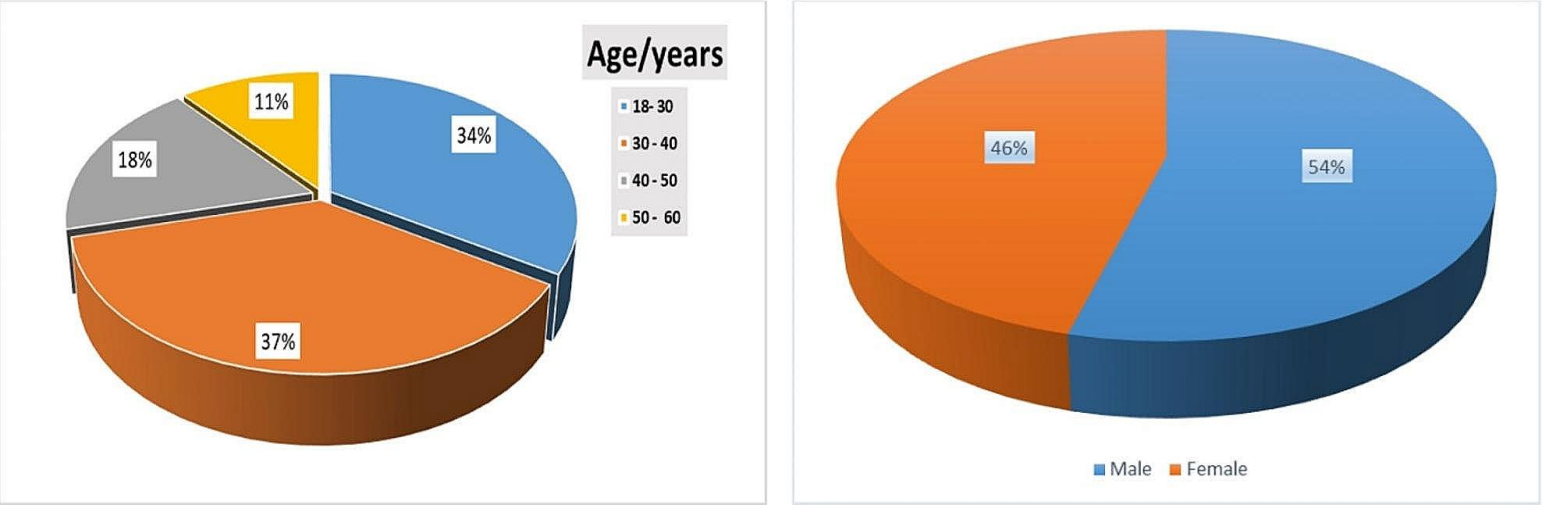
Percent of age and gender of subjects

**Table 1:** Relation between serum level of IL-17 and sociodemographic characteristics of the studied subjects (*n* = 100)

	IL-17 (pg/mL)	Test of significance
Age/years		
18–30	57.5 (14–210) (32.5–76.5)	KW = 6.18
30–40	55 (15–160) (32.0–73.0)	*P* = 0.103
40–50	64.5 (15–502) (45–82)	
50–60	78 (14–260) (63–86)	
Gender		
Male	62 (14–260) (39.5–78.5)	Z = 0.01
Female	60.5 (14–502) (44.75–81.25)	*P* = 0.992
Residence		
Urban	61.5 (14–502) (36.75–78.5)	Z = 0.434
Rural	62 (15–260) (41.25–79.5)	*P* = 0.664
Special habits		
Smoking	62 (16–260) (40.25–79)	Z = 0.108
No	61.5 (14–502) (39.5–80)	*P* = 0.914
Surgical history		
Yes	68 (14–210) (60.5–92.75)	Z = 1.72
No	59.5 (14–502) (35-77.25)	*P* = 0.085
Diabetes mellitus		
Yes	78 (14–95) (18–90.5)	Z = 0.316
No	62 (14–502) (42–78)	*P* = 0.752
Hypertension		
Yes	75 (62–86) (64.5–84)	Z = 1.38
No	60.5 (14–502) (36.5–79.5)	*P* = 0.167

IL-17 described as median (min - max) (interquartile range), KW: Kruskal Wallis, Z:Mann Whitney U test*statistically significant

Table (1) showed a non-statistically significant relation between serum level of IL-17 and sociodemographic characteristics including age, sex, residence, smoking habit, surgical history, presence of diabetes, hypertension (*p* > 0.05).

### Comparison between cases and control

In our study, 69.6% of cases (55 patients) were UC with segment distribution (26.6% left-sided, 24.1% recto-sigmoid and 18.9% extensive pan-colitis by colonoscopy). 30.4% (24 patients) were CD (18 cases were inflammatory, 4 cases werestricturing and 2 cases were fistulizing). All the studied UC cases showed activity by Mayo score; 94.5% moderate to severe, while 5.5% were mild active cases.CD cases (95.9%) showed activity on CDAI, while (4.1%) were of score ˂ 150 by CDAI.

In Table (2) there was statistically significant higher median IL-17 detected among cases than control group (65 versus 19).Mean hemoglobin level was lower among cases than control group(10.55 versus 11.52, respectively)(p ˂0.05).

**Table 2:** Comparison of different biochemical markers between cases of IBD& control groups

	Cases group *n* = 79	Control group *n* = 21	test of significance
IL-17 ( pg/mL)	65(21–502)	19(14–35)	Z = 6.92 *P* < 0.001*
WBCs (/mm3)	7.5 [–]	8 [–]	Z = 0.845 *P* = 0.398
Hb (gm/dl)	10.55 ± 1.31	11.52 ± 0.99	t = 3.25 *P* = 0.001*
Platelet (/mm3)	275.53 ± 75.67	272.24 ± 68.46	t = 0.263 *p* = 0.793
CRP (mg/dL)	24(4-131)	18(12–48)	z = 0.04 *p* = 0.965
ESR (mm/hour)	15(5-100)	19.5(12–25)	z = 0.120 *p* = 0.904

t: Student t test, Z: Mann Whitney U test, *statistically significant

### Relation between serum IL17 and IBD cases

Table (3) showed a statistically significant association between IL-17and type of disease. Median IL-17 was higher among UC than CD (71 & 55 pg/ml, respectively; *p* = 0.002).

**Table 3:** Relation between IL-17 and UC & CD cases

	IL-17 (pg/mL)	Test of significance
Ulcerative colitis (UC)	71 (21–502 ) (60–84)	z = 3.04
Crohn’s disease (CD)	55.0 (25.0–260) (45.5–73)	*P* = 0.002*

IL-17 described as median (min - max) (interquartile range), KW: Kruskal Wallis test

*statistically significant

Table (4) showed median IL-17 was higher among left sided UC and extensive UC than recto-sigmoid UC (78, 77 vs. 65 pg/ml respectively; *p* < 0.05).There was a statistically significant association between IL-17 and CDphenotype(*p* = 0.034). Median IL-17 was higher among fistulizing than stricturing and inflammatory (171, 62 & 53 pg/ml, respectively).

**Table 4:** Relation between IL-17 and UC extension by colonoscopy and CD phenotypeamong the studied IBD cases

Extension by colonoscopy	IL-17 (pg/mL)	Test of significance
Recto-sigmoid UC	65 ( 52–201 ) ( 60–78)	KW = 10.22
Left sided UC	78 (21–502) (58.5 - 87.5 )	*P* = 0.017*
Extensive UC	77 (51–210) (61.5–84.5)	
ILeo-cecal UC	55.0 (25.0–260) (45.5–73)	
Crohn’s disease type		
Inflammatory	53 (25–80) (42–66.75)	KW = 6.77
Stricturing	62 ( 55–73) (56.75–70.25)	*P* = 0.034*
Fistulizing	171 (82–260) (82–260)	

IL-17 described as median (min - max) (interquartile range), KW: Kruskal Wallis test *statistically significant

### Relation between IL17 and lines of management of IBD

All cases of IBD received treatment in the form of 5- aminosalicylic acid (5-ASA), while 54.4% used oral or systemic glucocorticosteroids plus azathioprine,29.1% of cases required biological treatment as anti TNF (Infliximab, Adalimumab) or anti-interleukin IL-12 and IL-23 antibody (Ustekinumab).

There was no statistically significant association between treatment lines and IL-17 (*p* = 0.712). Median IL-17 was 70 pg/ml among cases with biological treatment, 65 among cases with azathioprine & steroid treatment and 60 among cases with 5 - ASA treatment.

Table (5) showed thatthere was a non-statistically significant association between median serum level of IL-17 & duration of disease (*P* = 0.6).However there was a statistically significant association between serum IL-17 level and cases required hospital admission. Median IL-17 was higher among hospitalized cases than non-hospitalized (73 & 55, pg/ml respectively; *p* < 0.002).

**Table 5:** Relation between median IL-17, lines of treatment, duration of disease and hospital admission among IBD cases

	IL-17 (pg/mL)	Test of significance
Treatment lines		
5- ASA	60 (51–502)	KW = 0.679
Azathioprine + Steroid	65 (21–210)	*P* = 0.712
Biological lines	70 (25–260)	
Duration of disease in years		
˂ 1 year	55 (15–260)	KW = 0.886
1–5 years	55.5 (14–502)	*P* = 0.642
5–10 years	61.5(16–201)	
˃ 10 years	72 (14–160)	
Hospital admission		
Yes	73 (41–260)	Z = 3.15
No	55 (14–502)	*P* = 0.002*

IL-17described as median (min-max), KW: Kruskal Wallis test, Z:Mann Whitney U test*statistically significant

### Analytical data

Table (6) showed a statistically significant negative correlation between hemoglobin level and IL-17 (*r*= -0.281, *p* = 0.005). However, a non-statistically significant correlation was detected between IL-17 and all other laboratory findings as CRP, ESR, WBCs and fecal calprotectin (*p* > 0.05).

**Table 6:** Correlation between different laboratory parametersand IL-17 among IBD cases

	R	P-value
WBCs	0.09	0.374
Haemoglobin (Hb) (gm/dl)	-0.281	0.005*
Platelet	0.053	0.599
CRP	-0.101	0.363
ESR	0.094	0.396
Calprotectin	0.069	0.544

R: Spearman correlation co-efficient, *statistically significant

Area under curve was excellent for differentiating between cases of IBD and control group with area under curve = 0.993 by 95% CI (0.981-1.0). The best detected cut off point from curve was 32pg/mL yielding sensitivity of 97.5% and specificity of 95.2%.

**Fig. 2:**
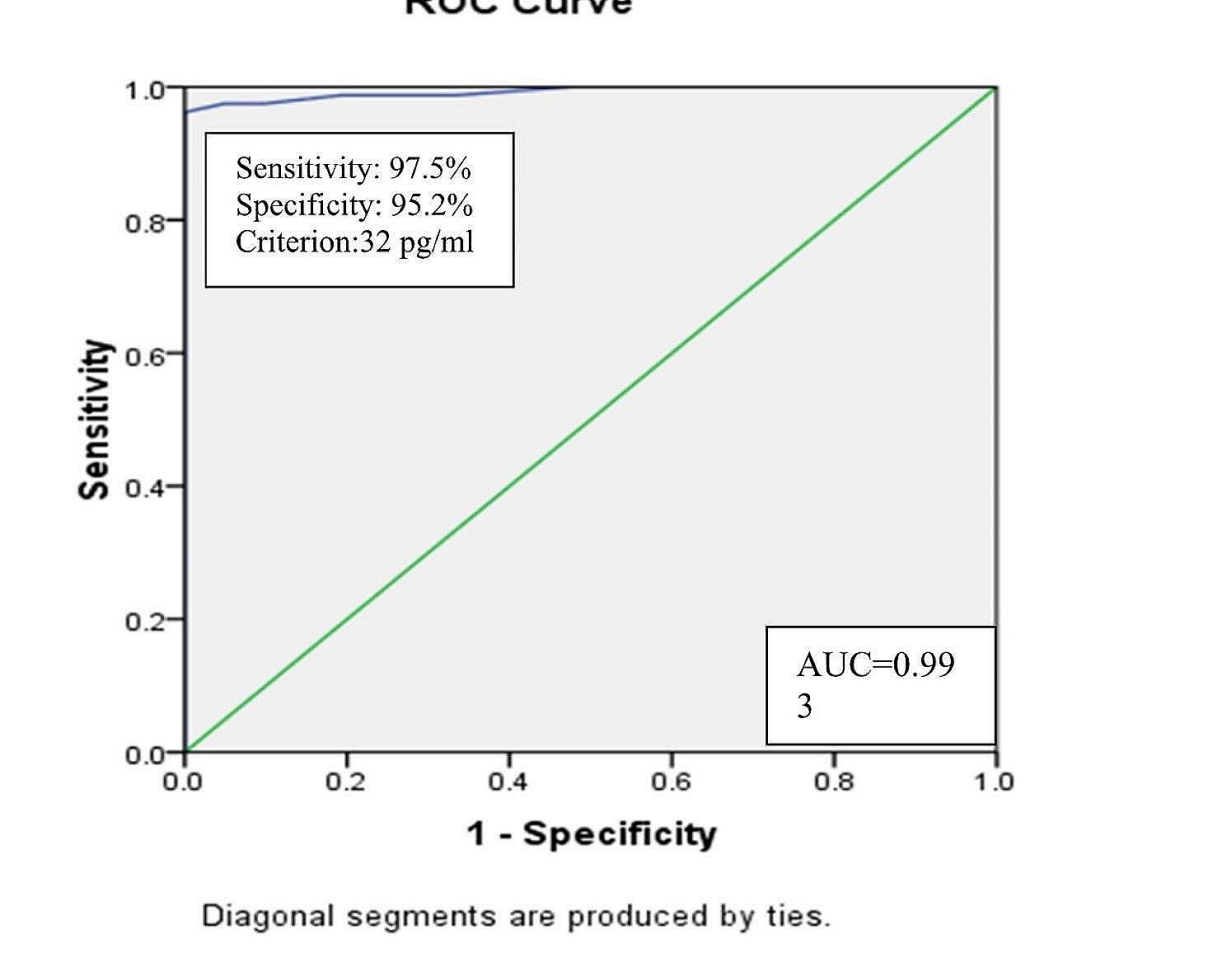
Cut off value by ROC curve of serumIL-17 in differentiating cases and control groups

**Table 7:** Validity of IL-17 in differentiating activity of CD &UC

	AUC (95% CI)	P value	Cut off point	Sensitivity%	Specificity%
In differentiating between moderate activity vs. other activities assessed by CDAI of CD					
IL-17	0.654 (0.427–0.880)	0.202	54.5	61.5	63.6
In differentiating between moderate to severe from mild activity assessed by Mayo score of UC					
IL-17	0.565 (0.407–0.723)	0.431	68.0	52.6	58.3
In differentiating between cases & control					
IL-17	0.993 (0.981-1.0)	< 0.001*	32.0	97.5	95.2

AUC: Area Under curve

## Discussion

IL-17 is a pro-inflammatory cytokine which is involved in triggering strong immune responses during chronic inflammation.It has been implicated in several inflammatory autoimmune disorders, such as rheumatoid arthritis and systemic lupus erythematosus [9].

Th17 cells have been considered as novel targets for disease activity monitoringwith therapeutic implications. Serum level of IL-17 could become a valuable biomarker for assessing disease severity subtypes in both CD and UC, by testing it against other biomarkers–which are frequently used in clinical practice- such as CRP, ESR and fecal calprotectin [10].

Regarding the relation between IL-17 and sociodemographic characteristics of the studied cases, there was no significant relation between median serum level of IL-17 and sociodemographic characteristics including age, sex, residence, or special habits (*p* > 0.05). Most of patients included in this study (78.5%) were non-smokers. See Table **(1).**

While, in another study theyfound that mean serum level of IL-17 was significantly increased in smoker UC patients compared with non-smokers ± SD (51.9 ± 19.4 vs. 31.6 ± 25.5 pg/ml; respectively, *p* = 0.022). In addition, smoker CD patients showed a significantly increased mean serum level compared to non-smokers (72.7 ± 28.5 vs. 52.2 ± 22.6 pg/ml; respectively, *p* = 0.038), there was also significantly increase in mean serum level of IL-17 among UC males compared to females (57.3 ± 18.2 vs. 34.5 ± 22.5 pg / ml; respectively *p* = 0.005) [4].Most of the patients included were smokers (around 55%)also this was just an observation with no supported evidence in literature.

In Table **(2)**:themedian of IL-17 was statistically significant higher among cases than control group (65 versus 19 pg / ml, respectively). Mean hemoglobin level was lower among cases than control group ± SD (10.55 ± 1.31 versus 11.52 ± 0.99 gm / dl, respectively) because most of our cases were in activity by Mayo score and CDAI with recurrent bloody diarrhea.This comes in agreement with several studies that showed significant elevation of IL-17 level in the IBD patients either UC or CD than in the control group [11, 12].

On the other hand, ***Sahin et al., 2014*** found that there was neither significant difference in the mean serum IL17 levels between active CD and healthy subjects ± SD ( 24.29 ± 11.03 versus 27.93 ± 12.07,pg/ml, respectively) nor between active and quiescent CD patients ( 23.82 ± 11.12 versus 24.61 ± 11.06 pg/ml, respectively) [7].In their study serum level of IL-17 was detectedfor 50 CD subjectsin comparison to 40 healthy controls. Twenty CD patients only were active while the rest were quiescent. This may explain why serum IL-17 showed near levelswith no significant difference among groups. Also intheir study there were no UC cases represented in sample size.

Moreover, the current study - Tables **(3,4)-**showed a statistically significant association between IL-17, type and distribution of disease.Lucaciu L. et al.,2021found that serumIL-17 levels were significantly higher in severe active UC patients than CD [12].On the other hand, another study showed that the level of IL-17–tested in 30 UC and 30 CD patients- had neither significant variation between UC and CD patients (*p* = 0.7),nor difference between CD patients according to disease location and behavior (*P* > 0.05) [4]. May be because most of UC cases (80%) were left sided with or without proctosigmoiditis and 80% of CD cases were just ofinflammatorytype; so limited percent were with pan-colitis or complication of IBD. Besides, most of patients recruited were treatment responsive.

On testing the relation of different treatment lines and IL-17, we found that there was no statistically significant association between treatment lines and IL-17 (*p* = 0.712).However there was a statistically significant association between serum IL-17 level and cases required hospital admission either due to recurrent exacerbation orfor management of complications.See Table (5). This may be attributed to excess release of cytokines including IL-17.

According to disease activity, there was a non-statistically significant association between IL-17 and Mayo scoreof UC activity (*p* = 0.4) with higher median IL-17 among moderate to severe active cases.Also, there was a non-statistically significant association between IL-17 and CDAI. Some studiesexplored the serum level of pro- and anti-inflammatory cytokines (including IL-6, IL-10, IL-13, IL-17, TNF-α and TGF-β) in the patients of UC and IBS. There was not significant correlation between cytokines level (IL-17) and Mayo score in UC [13] and between IL17 and CDAI [7].In contrast, other studiesshowed that there was a positive correlation between IL-17 in the IBD group and endoscopic activity index in UC and CD in comparison to a relatively matched large control group [14].There wasconfirmation by tissue sample showing more IL-17 expression in the mucosa and serum in IBD patients than control mostly related to altered immune and inflammatory responses in the intestinal mucosa [15].

As for the disease behavior, there was a statistically significant association between IL-17 and CD phenotype(*p* = 0.034). IL-17 was significantly higher in CD patients who developed intestinal complications, such as fistulae, abscesses, and need for surgery at a certain point in time [12].These patients suffer more inflammation with altered immunity.

The current study illustrated that area under curve (AUC) was poor for IL-17 in differentiating between moderate activity vs. other activities assessed by CDAI (AUC = 0.654) yielding sensitivity of 61.5% and specificity 63.6%. Similarly, for UC by Mayo score (AUC = 0.565) yielding sensitivity of 52.6% and specificity 58.3%. However it was excellent for differentiating between cases of IBD and control group (AUC = 0.993) with the best detected cut off point from curve is 32 yielding sensitivity of 97.5% and specificity of 95.2%.As in Fig. (2) and Table (7).

Similarly serum IL-17 level was not a good indicator for differentiating active CD from inactive disease, and can not be considered as an accurate marker for monitoring the disease activity [7].May be IL-17 producing inflammatory cells were effectively controlled by immunosuppressive drugs given to patients at point of activity.AlsoAUC was poor for IL-17 in differentiating between moderate active CD vs. other activities assessed by CDAI (AUC = 0.667).But it was goodin differentiating between moderate to severe UC from mild active cases(AUC = 0.803) yielding sensitivity of 64.71% and specificity 100% [12].

Limitationsof our study were that ourresults were based only on a small number of patient sampleand at a single time-point analysis of serum IL-17.This may be related to its high cost and difficulty to provide in market. We measured onlyserum IL-17 by ELISA, without correlation to its histological level within the inflamed intestinal mucosa.However IBD patients were represented both UC and CD(with different disease location and behavior) and most of them were in activity of disease. Also patients were compared to matched demographic group of healthy controls.

In conclusion, serum IL-17 level is elevated in IBD patients but it is neither activitynor prognostic marker.Serum IL17 measurement is not a useful tool for detecting or monitoring IBD activity. The results of our study suggest a role for IL-17 in the etiology and pathogenesis of UC and CD.

## Data Availability

Data were collected from the registered medical records following the institutional ethics committee and agreement with the Helsinki Declaration of 1975, revised in 2008. You may contact [corresponding author / profatma2000@mans.edu.eg] if someone wants to request the data from this study.

## Declarations

### Confirmation

*All authors have confirmed the manuscript for submission in this journal.

*The content of this manuscript has not been published, or submitted for publication elsewhere.
